# Treat-to-Target in Ulcerative Colitis: How Soon Is Now?

**DOI:** 10.3390/jcm15020759

**Published:** 2026-01-16

**Authors:** Laura Parisio, Giuseppe Cuccia, Giuseppe Privitera, Federico Castaldo, Luigi Carbone, Laura Maria Minordi, Daniela Pugliese

**Affiliations:** 1 UOS Gastroenterologia, Ospedale Isola Tiberina Gemelli Isola, 00186 Rome, Italy; laura.parisio.fw@fbf-isola.it (L.P.); giuseppecuccia24@gmail.com (G.C.); 2 Department of Biomedical Sciences, Humanitas University, Pieve Emanuele, 20072 Milan, Italy; giuseppe.privitera@humanitas.it; 3 Dipartimento di Diagnostica per Immagini e Radioterapia Oncologica, Fondazione Policlinico Universitario “A. Gemelli” IRCCS, 00168 Rome, Italy; federico.castaldo8@gmail.com; 4 UOC Pronto Soccorso, Medicina d’Urgenza e Medicina Interna, Ospedale Isola Tiberina Gemelli Isola, 00186 Rome, Italy; luigi.carbone@fbf-isola.it; 5 UOC di Radioterapia Oncologica ed Ematologia, Dipartimento di Diagnostica per Immagini, Fondazione Policlinico Universitario “A. Gemelli” IRCCS, 00168 Rome, Italy; lauramaria.minordi@policlinicogemelli.it; 6 Department of Translational Medicine and Surgery, Università Cattolica del Sacro Cuore, 00168 Rome, Italy; 7 IBD Unit-CEMAD (Centro Malattie Apparato Digerente), Medicina Interna e Gastroenterologia, Fondazione Policlinico A. Gemelli IRCCS, 00168 Rome, Italy

**Keywords:** treat-to-target, ulcerative colitis, biomarkers, ultrasound, endoscopy

## Abstract

Ulcerative colitis (UC) is a chronic progressive inflammatory bowel disease, with evolutive potential for extension to the entire colon, development of complications and need for colectomy. Therapeutic goals in UC have moved from symptom control to more stringent outcomes such as endoscopic and histologic remission, which have been observed to correlate with improved long-term outcomes. Disease clearance, a composite endpoint simultaneously including clinical remission, endoscopic and histologic healing, has been recently proposed as the ultimate target. A treat-to-target approach, as endorsed by the STRIDE II consensus, with a tight monitoring and treatment escalation when predefined endpoints are not reached, is proposed as a strategy to achieve complete disease control. However, unlike Crohn’s disease (CD), the evidence supporting this approach for the management of UC is limited and its implementation in routine clinical practice is not widely diffused. Recent real-life data show that almost half of UC patients are not adequately controlled with current therapies according to STRIDE II criteria, due to steroid overuse, persistent signs of inflammation, active extra-intestinal manifestations and impaired quality of life. This perspective paper explores current evidence and future directions on treat-to-target strategies in UC for clinical research and practice.

## 1. Introduction

Ulcerative colitis (UC) and Crohn’s disease (CD) are chronic inflammatory bowel diseases (IBDs) usually requiring life-long treatment to maintain symptomatic remission and to prevent the development of complications. Modern chronic IBD management has undergone a profound paradigm shift, evolving from reactive, symptom-based care to proactive strategies focused on long-term disease control and improved patient outcomes. Indeed, the correlation between symptoms and intestinal inflammation is inconsistent, and patients in clinical remission may still have underlying endoscopic and histological disease activity, which is associated with a higher risk of adverse outcomes [1,2].

The treat-to-target (T2T) model, which advocates for predefined outcome thresholds and proactive treatment escalation to achieve them, originated in the cardiovascular field, wherein treatment strategies for conditions like hypertension and diabetes mellitus are guided by specific target values, such as blood pressure or hemoglobin A1c levels, respectively.

This approach was subsequently adopted by rheumatologists in the management of rheumatoid arthritis, following studies that demonstrated the benefits of intensive disease activity monitoring, treatment adjustment, and early intensive treatment in achieving superior outcomes compared to standard care [3,4,5].

About IBD, the Selecting Therapeutic Targets in IBD STRIDE II Consensus, embodies the abovementioned shift by advocating for predefined therapeutic goals, tight monitoring, and timely treatment adjustment when those goals are not met.

Symptomatic response and remission, defined as ≥50% decrease or resolution of rectal bleeding and normalization of stool frequency for UC and abdominal pain and diarrhea for CD, are key goals for patients and crucial short-term and intermediate targets, respectively. Normalization of C-reactive protein (CRP) and reduction in fecal calprotectin (FCP) have emerged as important intermediate endpoints that can inform treatment optimization.

Long-term therapeutic objectives include endoscopic healing, normalization of quality of life, normal growth in children and absence of disability, aiming for more stringent and sustainable disease control. Additionally, the STRIDE II consensus proposes histologic and transmural healing as adjunctive measures for UC and CD, respectively, although they are not formal treatment targets.

Despite these recommendations advocating for the pursuit of more stringent endpoints in the management of IBD, it is a matter of fact that the direct evidence supporting T2T management, particularly regarding treatment escalation guided solely by biomarkers, and its long-term effectiveness, is limited, especially for patients with UC.

A recent survey by the European Crohn’s and Colitis Organization (ECCO) revealed significant variability in the management of IBD and implementation of T2T in real-world settings. Notably, only a quarter of healthcare providers routinely perform an endoscopy after initiating an advanced therapy to monitor disease activity [6]. Furthermore, the PODCAST-IBD study found that approximately half of patients have a suboptimal disease control according to STRIDE II Consensus Recommendation with consistent results across countries [7,8,9].

This perspective paper discusses the evidence and future perspectives for implementing T2T approaches in UC, with implications for clinical trials and real-world patient care.

## 2. Evolution of Targets in UC

In the very earliest randomized controlled trials (RCTs) in UC, investigators focused on assessing immediate and measurable, such as relief of bleeding, reduction in stool frequency, abdominal pain, and other patient-reported clinical features.

The 1955 trial by Truelove and Witts established that corticosteroids induce rapid and dramatic clinical improvement and reduce short-term mortality in severe acute colitis, thereby anchoring symptomatic control as the first demonstrable trial endpoint in IBD [10]. Later on, clinical response and remission, that is improvement and resolution of symptoms, respectively, induced by corticosteroids therapy, emerged also as primary outcomes for studies in CD [11].

However, those landmark studies also revealed a crucial limitation that persists in modern guises: clinical remission does not necessarily equate to absence of underlying intestinal injury [12]. Furthermore, they highlighted the risk of development of steroid dependence and subsequent relapses as unresolved problems, underscoring the need for endpoints and therapies that can achieve sustained disease control and minimize steroid use [13,14,15].

For years, thiopurines were the recommended maintenance therapy for steroid-dependent patients, as real-world studies and small RCTs demonstrated their effectiveness, primarily in achieving clinical remission and to some extent, endoscopic remission, although these studies are limited by short follow-up periods and heterogeneous patient populations.

The biological era inaugurated a methodological shift. Anti TNF-alpha agents and later other mechanism-targeted drugs were tested in phase 2 and 3 trials that routinely incorporated objective measures of mucosal inflammation—first endoscopic appearance, and later biomarkers and histology- alongside traditional clinical indices. Moreover, modern trials have implemented measures to reduce variability, introducing central review of endoscopic imaging and centralized analysis of laboratory samples, to reduce inter-observer bias and standardize cut-offs and techniques for laboratory tests.

The Active UC Trials (ACT 1 and ACT 2), the first RCT with biologics in UC, exploring the efficacy of Infliximab for induction and maintenance of remission, included mucosal healing, defined as an absolute Mayo endoscopic subscore (MES) of 0 or 1, as secondary endpoints [16].

Subsequently, population-based and real-world studies provided evidence supporting the positive association between mucosal healing and long-term favorable disease course. A recent meta-analysis of 43 studies, including over 17,000 patients, found that achieving mucosal healing was associated with a significant reduction in the risk of clinical relapse, hospitalization, and surgery. However, the results were limited by substantial heterogeneity among studies and inconsistent definitions of mucosal healing [17].

Indeed, various scoring systems and cut-off values have been proposed to measure endoscopic activity across studies. The MES is the most widely adopted in both clinical trials and daily practice, with endoscopic remission typically defined as either normal mucosa (MES 0) or decreased vascular pattern, mild erythema and mild friability (MES 1) [18,19].

However, whether achieving a more stringent endoscopic target of MES 0 is associated with improved long-term outcomes remains a topic of debate. A recent meta-analysis by Viscido et al. found that, among patients in steroid-free clinical remission, those with MES 0 had a reduced risk of clinical relapse, but not of hospitalization or colectomy, over a 12-month follow-up period [20]. Similar findings emerged from another metanalysis, showing that achieving MES 0 was associated with up to 50% lower risk of clinical relapse compared to MES 1. Notably, among patients with MES 0, achieving histologic remission was associated with further 63% reduction in relapse risk, resulting in an annual relapse rate of just 5.0% [21].

Histological resolution of inflammation emerged as a further evolution of targets, associated with reduced risk of long-term clinical relapse, UC-related hospitalization and cancer [22,23].

The importance of histologic healing was further underscored by the Ustekinumab’s pivotal trial UNIFI in UC, which expanded on this concept by prespecifying histologic remission as a measurable outcome. Notably, the trial included histo-endoscopic mucosal healing, a composite endpoint that required both endoscopic improvement and histologic normalization (defined as neutrophil infiltration in <5% of crypts, no crypt destruction, and no erosions, ulcerations, or granulation tissue) as secondary endpoints [24].

Subsequent RCTs, such as the U-ACHIEVE and U-ACCOMPLISH programs evaluating Upadacitinib, as well as the LUCENT study on Mirikizumab, have incorporated composite endpoints combining endoscopic and histologic remission, such as histo-endoscopic mucosal improvement (HEMI) and histo-endoscopic mucosal remission (HEMR), to provide a more comprehensive assessment of mucosal recovery [25,26].

However, despite these advances in RCT design, the actual benefit of achieving more stringent endoscopic and histologic remission remains understudied in a real-world setting. Furthermore, the lack of standardized definition of histological remission and variability in pathological reporting hinder its practical application as therapeutic target in clinical practice.

Moreover, histologic healing, while indicative of deeper mucosal resolution, may not necessarily translate into full symptomatic remission, implying that persistent bowel dysfunction may stem from non-inflammatory alterations.

Indeed, Colombel et al. analyzed data from an observational study, demonstrating that patient-reported rectal bleeding correlated more closely with endoscopic healing than abnormal stool frequency, which often persisted despite complete endoscopic and histologic remission [27]. Notably, the inclusion of histologic remission did not enhance concordance between clinical symptoms and mucosal healing. Furthermore, biomarker values (both FCP and CRP) showed no significant differences between endoscopic and combined endoscopic–histologic remission.

The evolution in UC of treatment targets has progressed with two key concepts: (1) Disease clearance, defined as the simultaneous achievement of symptomatic remission (based on patient-reported outcomes), endoscopic healing, and histological healing; (2) Comprehensive disease control encompassing resolution of symptoms (including bowel urgency, stool frequency and abdominal pain), normalization of biomarkers, steroid-free clinical remission, endoscopic improvement or remission, histologic healing, control of extra-intestinal manifestations, and improvement in previously unaddressed domains such as sleep disturbance, fatigue and quality of life) [28].

Preliminary data from the VERDICT trials, as discussed in the following paragraph, suggest that disease clearance can be achieved through a T2T approach [29]. However, the long-term benefits of this approach over strategies targeting only symptomatic and endoscopic remission remain to be confirmed.

In the aforementioned LUCENT program, bowel urgency, assessed using the Urgency Numeric Rating Scale (UNRS), was included among secondary endpoints, making a significant innovation in RCT design. This symptom, frequently reported by patients even when rectal bleeding and stool frequency are controlled, is conversely one of the most debilitating manifestations of UC, profoundly impacting patients’ quality of life [26,30].

The assessment of quality of life in clinical studies dates back to the 1990s–2000s, when disease-specific instruments such as the Inflammatory Bowel Disease Questionnaire (IBDQ) and its abbreviated form, the Short IBDQ (SIBDQ), were developed and validated [31,32]. More recently, fatigue has been increasingly evaluated with tools like FACIT-F; meaningful reductions in fatigue scores reflect substantial improvements in patient well-being [33].

Multiple studies and systematic reviews have demonstrated that better endoscopic and histologic disease control correlates with improved health-related QoL [34]. Based on these findings, quality-of-life measures have been increasingly incorporated into clinical trials as primary or co-primary endpoints, complementing traditional clinical and endoscopic outcomes.

**Figure 1:**
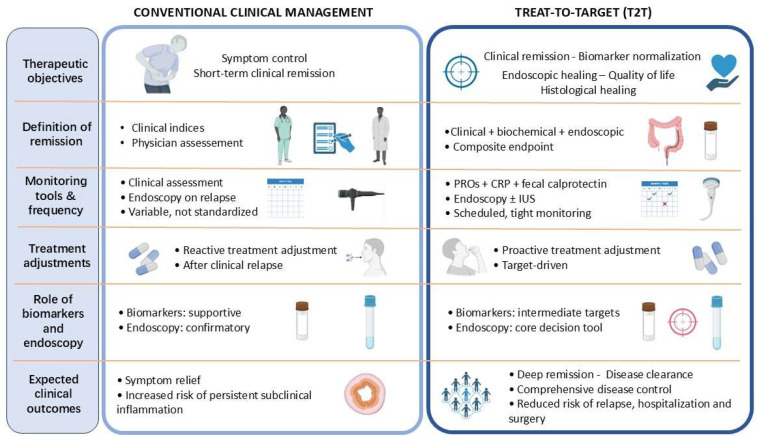
Conceptual comparison between conventional symptom-driven management and the treat-to-target approach in inflammatory bowel disease. PROs: Patient Reported Outcomes; CRP: C-Reactive Protein; IUS: Intestinal Ultrasound.

Building on these grounds, the T2T approach marks a paradigmatic shift in the clinical management of patients with IBD, moving beyond the traditional approach driven by symptoms. Figure 1 shows a conceptual comparison between conventional symptom-driven management and the T2T approach in IBD, highlighting differences in monitoring strategies, therapeutic decisions, and long-term outcomes.

## 3. What We Already Know on T2T

### 3.1. T2T Treatment Strategies in CD

With regard to CD, four major T2T strategy studies have been published to date, each adopting a different treatment target: REACT1 (clinical), CALM (biomarkers + clinical), REACT2 (endoscopic), and STARDUST (endoscopic, including ultrasound assessment).

The REACT1 study was the first to evaluate the validity of a T2T strategy for CD management [35]. This open-label, cluster-randomized controlled trial compared an early combined immunosuppression (ECI) approach—Adalimumab plus an immunosuppressant with the conventional step-up therapy. In both groups, patients were re-evaluated every 12 weeks using the Harvey–Bradshaw Index (HBI), and treatment was escalated in the absence of clinical remission (HBI ≥ 4). The study included CD patients naïve to advanced therapies, balanced for demographic and disease characteristics, with 1084 enrolled in the ECI arm and 898 in the conventional management group. After 24 months of follow-up, there were no significant differences in clinical remission rates between the two groups. However, the ECI strategy proved significantly more effective in reducing serious disease-related complications, hospitalizations, and surgeries compared with conventional management (HR = 0.73, 95% CI = 0.62–0.86, *p* = 0.0003).

The subsequent CALM study—an open-label, phase 3, multicenter randomized controlled trial—further advanced the T2T strategy by integrating clinical assessment with biomarker-based monitoring [36]. A total of 244 patients with early CD (non-complicated, naïve to advanced therapies, and with short disease duration) were enrolled. After a standard 8-week course of corticosteroids, patients were randomized into two groups: (1) The clinical management group, in which initiation and optimization of Adalimumab therapy and potential addition of azathioprine were guided solely by clinical activity (assessed using the CD Activity Index [CDAI]) and prednisone use; (2) the tight control group, in which treatment decisions were based on both clinical parameters and inflammatory biomarkers—specifically CRP and FCP.

Patients were reassessed every 12 weeks. After 48 weeks, a significantly higher proportion of patients in the tight control arm achieved endoscopic remission (46%), defined as a CDEIS score < 4 and absence of ulcers, compared with those in the clinical management arm (30%; *p* = 0.010). Importantly, no significant differences in safety profiles were observed between the two groups. Moreover, patients achieving deep remission-defined as endoscopic remission with no steroid use for more than 8 weeks- have a lower risk of long-term disease progression (defined as development of new internal fistulas or abscesses, strictures, perianal fistulas or abscesses, or the need for hospitalization or surgery for CD) [37]. Although conducted in a population with relatively early-stage disease, the CALM study underscored the value of a comprehensive, biomarker-guided approach to disease monitoring, rather than relying solely on clinical symptoms.

In 2022, at the United European Gastroenterology Week, the preliminary results of the REACT-2 study—a multicenter, cluster-randomized controlled trial—were presented [38]. Like the REACT-1 study, patients were randomized to either a conventional step-up approach with treatment escalation aimed at achieving clinical remission (HBI ≤ 4) vs. an early combined immunosuppression (ECI) strategy, but, in this case, with treatment escalation targeting the absence of endoscopic ulcers (>5 mm). After 24 months, no significant differences were observed between the two strategies regarding the risk of hospitalizations, surgical or non-surgical complications, CD–related medications, and procedure-related adverse events. However, among patients with active disease at baseline—defined by CRP > 5 mg/L and the presence of endoscopic ulcers—the endoscopy-driven strategy significantly reduced the risk of complications by 29% compared with the clinically driven approach (*p* < 0.001). These findings further support the importance of a treatment strategy focused on mucosal healing, particularly for patients with more active or severe CD.

Finally, the STARDUST study, a multicenter, randomized, open-label phase 3b trial, introduced a treatment strategy that integrates clinical, biochemical, and endoscopic parameters to guide therapeutic decisions [39]. A total of 440 patients with CD responders to Ustekinumab induction therapy at week 16 were randomized to two treatment arms: the Standard of Care (SoC) arm, in which Ustekinumab dose optimization from 12- to 8-week intervals was based solely on the CDAI score; and the T2T arm, in which further optimization to every-4-week dosing was permitted based on combined clinical, biochemical, and endoscopic responses.

The study population consisted of patients with long-standing CD (mean disease duration 7.7 years in the T2T group and 5.9 years in the SoC group); roughly 62% had previously received at least one advanced therapy.

No significant difference in endoscopic response (defined as a ≥50% reduction in the SES-CD score from baseline), was observed between the two strategies (38% in T2T vs. 30% in SoC; *p* = 0.087). However, when patients who discontinued treatment before week 48 for reasons unrelated to drug efficacy were excluded, a significant difference emerged (40% in T2T vs. 31% in SoC; *p* = 0.046). Although the primary endpoint was not met, the STARDUST trial underscored the value of a management strategy that integrates clinical, biochemical, and endoscopic data, suggesting that a tight control approach may represent an important step toward more effective personalized therapy in CD.

In a substudy of the STARDUST that included ultrasound assessments at weeks 0, 4, 8, 16, and 48 in 77 patients, a progressive improvement in ultrasound response and transmural remission was observed, consistent with the endoscopic findings. A 90% concordance between the two assessment methods was recorded at baseline, with a negative predictive value of 73% for the absence of ultrasound response at week 4 predicting the lack of endoscopic response at week 48, highlighting ultrasound’s potential as an early monitoring tool for therapeutic response [40].

### 3.2. T2T Treatment Strategies in UC

Evidence supporting T2T is considerably more limited in UC.

Several factors may account for this gap. First, UC has historically been viewed as a disease with more easily assessable activity, in which clinical symptoms often show a closer correlation with mucosal inflammation. This perception may have reduced the perceived need for rigorous, objective monitoring strategies. In addition, the natural history of UC—characterized by continuous inflammation confined to the colon—has traditionally favored step-up therapeutic approaches, thereby delaying the widespread adoption of early intensive treatment strategies. By contrast, CD is defined by transmural inflammation, progressive structural damage, and a higher risk of irreversible complications, features that have promoted earlier acceptance of proactive treat-to-target paradigms. Finally, from a methodological perspective, many UC clinical trials have prioritized short-term clinical and endoscopic outcomes rather than long-term disease-modifying endpoints.

In 2014, Osterman et al. conducted an open-label, randomized trial involving 52 patients with UC who were in clinical remission on mesalamine 2.4 g/day but had elevated FCP (≥50 µg/g) [41]. Participants were randomized 1:1 to either continue the same dose or escalate to 4.8 g/day. The primary endpoint (FCP < 50 µg/g without relapse at 6 weeks) was achieved by a significantly greater proportion of patients in the dose-escalation group compared with the stable-dose group (26.9% vs. 3.8%, *p* = 0.049). Additionally, a nearly significant greater reduction in median FCP levels, adjusted for baseline values, was observed with dose escalation (−70 µg/g [IQR −378 to 4] vs. −11 µg/g [IQR −231 to 101], *p* = 0.06). Interestingly, there was no difference between the two groups in time to clinical relapse over 48 weeks. However, among patients in remission at week 12, those with FCP ≥ 200 µg/g experienced a significantly shorter time to clinical relapse than those with FCP < 200 µg/g (*p* = 0.01), with a similar trend for FCP ≥ 100 vs. < 100 µg/g (*p* = 0.09). These findings reinforce the notion that achieving biochemical disease control is associated with improved long-term outcomes, regardless of the specific therapeutic strategy used to achieve it.

A subsequent open-label, randomized study published in 2015 provided consistent results [42]. Patients with UC in clinical remission on low-dose aminosalicylates but with at least one flare during the previous year were randomized (3:2) to a T2T arm (wherein mesalamine dose was optimized if monthly FCP exceeded 300 µg/g; n = 51) or a control group without FCP-guided optimization (n = 40). Although the overall proportion of patients experiencing at least one relapse during the 18-month follow-up was numerically lower in the T2T group (35.3% vs. 50.0%, *p* = 0.23), this difference was not statistically significant. Notably, most relapses in the T2T arm (10 of 18) occurred in patients without preceding FCP elevation and thus without dose escalation. When comparing only those who underwent dose escalation in the T2T group with control patients who had FCP > 300 µg/g, a significantly lower relapse rate was observed in the intervention group (28.6% vs. 57.1%, *p* < 0.05). Collectively, these findings support the rationale for FCP-guided T2T optimization of mesalamine in UC, while also emphasizing the need to define optimal FCP thresholds for intervention. The ongoing VERDICT trial represents the first randomized controlled trial designed to assess T2T management with biologics in UC [29]. Patients with moderate-to-severe UC initiating Vedolizumab are randomized (2:3:5) to three strategies differing by their predefined treatment targets: (1) group 1: treat to symptoms (steroid-free clinical remission, SFCR); (2) group 2: treat to symptoms and endoscopy (SFCR + MES ≤ 1); (3) group 3: treat to symptoms, endoscopy and histology (SFCR + MES ≤ Geboes score < 2.0, that is disease clearance).

Patients are evaluated every 16 weeks (receiving clinical, biochemical, endoscopic, and histologic assessments), with Vedolizumab dose escalation permitted at weeks 16 and 32 if their assigned target is not achieved; by week 48, patients not meeting their target are managed at the investigator’s discretion. The primary endpoint is time from target achievement to UC-related complication among those who achieved their assigned target, over a follow-up over 96 months. Preliminary findings show that, among patients with observed data, 90.3% (84/93) in group 1, 77.3% (102/132) in group 2, and 67.2% (123/183) in group 3 achieved their group-specific target at week 48; in the intention-to-treat analysis, corresponding rates were 45.4% (84/185), 45.9% (102/222), and 46.4% (123/265). Notably, in group 3, a significantly higher proportion of biologic-naïve patients achieved the histologic-endoscopic target compared with previously exposed patients [43].

## 4. Intestinal Ultrasound in UC, a New Tool for T2T

Endoscopy remains the gold standard for assessing mucosal healing in UC, but its widespread use is hindered by its invasive nature, high costs and limited repeatability.

Recent findings have shown that UC is a transmural disease, involving not only the mucosa, but also deeper layers of the intestinal wall such as the muscularis mucosae and serosa [44]. As a result, there is growing interest in alternative diagnostic approaches, particularly intestinal ultrasound (IUS), which offers a noninvasive, bedside-repeatable, and potentially predictive tool for therapeutic response [45]. IUS is especially valuable in UC due to its ability to assess transmural inflammation, particularly in severe or long-standing disease, allowing for the evaluation of both structural and functional changes in the intestinal wall [46].

Emerging evidence supports the use of IUS as a noninvasive monitoring tool that complements clinical and biological assessments to evaluate disease activity in the short and intermediate term. In a T2T strategy, monitoring ultrasound parameters enables early therapeutic adjustments, reducing the risk of disease progression or treatment failure [47]. The sonographic parameters included: (1) Bowel wall thickness (BWT), whereas increased thickness strongly correlates with both endoscopic and clinical activity; (2) Wall stratification: where loss of normal stratification indicates active inflammation; (3) Doppler signal: where increase signal intensity is related to inflammation-induced hyperemia within the intestinal wall; (4) Extra-wall signs: such as presence of reactive lymph nodes and altered echogenicity of the surrounding mesenteric fat [48].

In a multicenter study, Maaser C. et al., consecutively enrolled 224 patients with active UC who had an increased BWT in the descending or sigmoid colon and initiated a new therapy according to physician judgment [45]. After 2 weeks, a significant decrease in the percentage of patients with increased BWT was observed compared to baseline (descending colon 83.0% to 42.9%; sigmoid colon 89.3 to 38.6%; *p* < 0.001 each) and this trend was sustained through week 6 and 12 (descending colon 43.4% and 37.6%; sigmoid colon 35.4% and 32.0%; *p* < 0.001 each). Additionally, a strong correlation was found between normalization of BWT and clinical response after 12 weeks of treatment, with 90.5% of patients with normalized BWT achieving symptomatic response compared to 9.5% without symptomatic response. (*p* < 0.001). Similarly, a single-center Italian study involving 49 patients initiating a new biological therapy, found that improvement in BWT at week 12, assessed using the Milan ultrasound criteria (MUC), was predictive of long-term endoscopic outcomes [49].

More recently, Dolinger and colleagues, conducted a study in a pediatric cohort of 42 patients starting advanced therapies, and found that achieving a BWT < 2.7 mm (OR 6.4; 95% CI, 1.8–27.0, *p* = 0.007), an absolute MUC < 6.0 (OR 5.7; 95% CI, 1.5–25.3, *p* = 0.015) and a FCP level < 177 mcg/g (OR 4.5; 95% CI, 1.1–23.6, *p* = 0.049) by week 8 were strong predictors of endoscopic remission within 12 months from baseline [50]. In contrast, the presence of inflammatory fat was negatively associated (OR 0.18 [95% CI, 0.04–0.75], *p* = 0.026).

Finally, trans-perineal ultrasound has also been proposed for early monitoring patients with UC. Specifically, improvement in rectal BWT as early as 1 week after starting advanced therapies has been associated with higher rate of clinical-endoscopic remission (assessed by Mayo score) and histo-endoscopic mucosal improvement (assessed by Geboes score) [51].

Thus, after initiating a new therapy, monitoring BWT in the early phase could be a practical, feasible and cost-effective approach, serving as a short-to-intermediate target to guide therapeutic decision-making in clinical practice. Moreover, advanced techniques such as contrast-enhanced ultrasound (CEUS) and elastography are emerging as promising tools to quantify perfusion and wall stiffness, with potential value as novel predictive endpoints [52,53].

However, some limitations remain in studying UC with IUS. It is operator-dependent, requires specific training, and currently lacks universally accepted cut-offs, even though several studies have attempted to validate scoring systems such as UC-IUS, MUC, and IBUS-SAS to promote standardization [45,48]. Integration with biomarkers and predictive algorithms based on artificial intelligence may further enhance the role of IUS as a central tool within the T2T, enabling a more personalized and dynamic approach to disease management [54].

IUS represents a promising tool in the T2T paradigm for UC, allowing frequent and noninvasive monitoring of therapeutic response. Sonographic parameters can serve as intermediate endpoints to guide treatment decisions, facilitating therapy escalation or adjustments before clinical relapse or irreversible structural damage occurs. To consolidate its role in clinical practice, the following are required: (1) Standardization of parameters and validation of scoring systems; (2) Multicenter studies to confirm the predictive value of IUS parameters; (3) Integration with biomarkers and advanced imaging techniques (e.g., CEUS, elastography). Although cost-effectiveness analyses in UC exist, including microsimulation models comparing symptom- and biomarker-based T2T strategies, formal economic evaluation of noninvasive monitoring tools such as IUS within T2T frameworks still lacks, despite their potential to reduce invasive procedures and resource utilization [55,56].

Nevertheless, IUS has the potential to become a cornerstone of T2T monitoring, optimizing clinical management, reducing the need for invasive procedures, and improving long-term patient outcomes.

## 5. Future Perspectives

A new concept that has emerged as a potential target for UC in the future is the prevention and arrest of fibrotic remodeling of bowel wall.

Although UC has traditionally been regarded as a mucosal disease, accumulating evidence indicates that fibrotic and structural remodeling of the colonic wall occurs, particularly in long-standing disease, and may contribute to symptoms and poorer outcomes [57,58]. Specifically, thickening of the muscularis mucosae and excessive extracellular matrix deposition in the submucosa have been observed, with deeper layers becoming affected only after profound ulceration of the submucosa; these changes correlate closely with the severity and chronicity of inflammation in UC patients [59,60].

Moreover, patients with long-standing disease exhibit thickening of the tunica muscularis, alterations in vascular network, and increased expression of markers like α smooth muscle actin, and collagen types I and III compared with patients with shorter disease duration or healthy controls [57]. The growing interest in fibrosis starts from the realization that fibrotic changes may represent a point beyond which mucosal damage becomes only partially reversible: even when inflammation is completely suppressed (clinical + endoscopic + histologic remission), residual fibrotic remodeling can sustain symptoms or impede full functional recovery. A study examining mucosal gene expression in patients who achieved mucosal healing after Infliximab treatment, revealed a persistent dysregulation of fibrosis-related mediators compared with healthy controls [61].

Over the past 2 decades, the TL1A/DR3 cytokine family has emerged as a crucial component of mucosal immunity and intestinal homeostasis.

Preclinical studies have identified TL1A as a key enhancer of immune activation during inflammation, and TL1A/DR3 signaling is increasingly recognized as a driver of intestinal fibrogenesis. In transgenic mice with constitutive TL1A expression in lymphoid or myeloid cells, increased collagen deposition and gut fibrosis were observed, accompanied by heightened activation of mucosal T cells and antigen-presenting cells [62]. Notably, blockade of TL1A with neutralizing antibodies reversed established colonic fibrosis and reduced the expression of profibrotic mediators such as connective tissue growth factor, TGF-β1, IL31Ra, and IGF-1, as well as decreasing fibroblast and myofibroblast numbers [63]. Primary intestinal myofibroblasts express DR3 and respond to TL1A stimulation by upregulating collagen and IL31Ra, directly linking this pathway to extracellular matrix production [64]. TL1A also promotes epithelial–mesenchymal transition through TGF-β1/Smad3, IL-13, and transcription factors such as ZEB1 and Snail1 [65]. Collectively, these findings establish TL1A/DR3 signaling as a central profibrotic axis and support the idea that its inhibition may simultaneously address inflammation and fibrosis.

Based on these preclinical data, monoclonal antibodies targeting TL1A have been developed in recent years. The first-in-human phase 1 study of the anti-TL1A “PF-06480605” in healthy volunteers demonstrated good safety and tolerability. The phase 2a TUSCANY trial evaluated PF-06480605 in moderate-to-severe UC and showed significant endoscopic improvement compared to the placebo, with acceptable safety profile [66]. Transcriptomic analysis of colonic biopsies and proteomic profiling of peripheral blood further confirmed PF-06480605–mediated inhibition of TL1A, showing downregulation of Th17- and fibrosis-related genes (IL-1B, IL-23A, IFN-γ, IL-21R) and decreased circulating IL-17A in responders [67].

Efficacy and safety of anti-TL1A antibodies were also assessed in the ARTEMIS-UC phase 2 trial (Clinicaltrials.gov: NCT04996797), which enrolled 135 UC patients and demonstrated markedly higher clinical remission rates at week 12 compared to placebo (26.5% vs. 1.5%, *p* < 0.001) [68]. While the antifibrotic potential of anti-TL1A therapy has been highlighted in preclinical work, fibrosis outcomes were not formally evaluated in these clinical trials. Notably, ARTEMIS-UC incorporated a genetic diagnostic tool to identify TL1A risk alleles, which correlated with improved treatment response and underscored the relevance of precision medicine approaches in IBD.

Several ongoing clinical trials are further evaluating TL1A inhibitors in UC and CD, including TUSCANY-2 (PF-06480605, phase 2b), APOLLO-CD (PRA023), TAHOE (RVT-3101, Clinicaltrials.gov: NCT05910528), and RELIEVE-UCCD (TEV-48574, a basket trial including both UC and CD, Clinicaltrials.gov: NCT05499130) [69,70]. These studies may ultimately establish TL1A inhibition as a dual anti-inflammatory and antifibrotic therapeutic strategy.

Unfortunately, as anti-TL1A therapies are still in the experimental phase and no head-to-head or real-world studies are currently available, it is not possible to propose an evidence-based positioning of these agents within the therapeutic algorithm.

However, one of the most challenging aspects of targeting fibrosis in UC lies in how to assess fibrotic changes beyond conventional histology, thereby allowing reliable monitoring of progression or regression.

Over the past decade, several imaging modalities, including ultrasound elastography, and advanced magnetic resonance techniques, have been extensively explored for the evaluation of intestinal fibrosis, nonetheless, none of these methods has yet been definitively validated as a reliable tool for quantifying fibrosis [71]. More recently, promising advances have emerged from the application of artificial intelligence (AI) to cross-sectional imaging, particularly through radiomics [72]. In parallel, biomarkers remain a focus of intensive research: specific microRNAs (miRNAs), circulating and intestinal proteins (collagen, ECM components, pentraxin-2, glycoproteins), enzymes (e.g., metalloproteinases), antimicrobial antibodies, growth factors like YKL-40, and gene variants have been examined [71].

Leucine-rich alpha-2 glycoprotein (LRG) is a promising biomarker for assessing inflammation in IBD. This protein released by hepatocytes, neutrophils, macrophages, and intestinal epithelial cells, in response to cytokine cascade, has shown potential in evaluating disease activity [73].

A recent Japanese study involving 79 patients who underwent multiple endoscopic examinations, measured LRG, CRP, and FCP levels to assess their correlation with endoscopic and histological disease activity. LRG levels correlated significantly with endoscopic activity (*p* < 0.0001 MES score), and with histological activity (*p* < 0.0001 Geboes score). LRG successfully identified patients with mucosal healing (MES 0–1, 69.9%) and histological remission (Geboes score < 2.1, 55.3%). Comparison with other biomarkers, LRG outperformed CRP but not FCP in predicting both endoscopic activity (areas under the curve [AUCs]: LRG 0.68, CRP 0.62, and FCP 0.79) and histological activity (AUCs: LRG 0.67, CRP 0.62, and FCP 0.79) [74].

Another molecule currently under investigation is the major urinary metabolite of prostaglandin E (PGE-MUM). PGE-MUM is a stable urinary metabolite derived from prostaglandin E2 (PGE2), a key mediator of inflammation with a very short half-life. Owing to its greater stability, PGE-MUM can be reliably measured and has emerged as a promising biomarker of inflammatory activity. In a prospective study, urinary PGE-MUM levels were assessed in 70 patients with UC in both clinical remission, defined by a Clinical Activity Index (CAI) ≤ 3, and endoscopic remission, defined as MES < 2 [75]. Over a 12-month follow-up period, 16 patients experienced clinical relapse, defined as an increase in the CAI of at least 4 points. Baseline PGE-MUM levels were significantly higher in patients who subsequently relapsed compared with those who remained in remission (*p* = 0.008; AUC 0.721, 95% CI 0.556–0.886).

More recently, an IgG autoantibody directed against integrin αvβ6 has been identified and shown to discriminate patients with UC from those with CD and from healthy controls. In an Italian study, serum anti-αvβ6 antibody levels were measured in 108 patients with UC, 103 patients with CD, and 62 healthy controls [76]. Elevated anti-αvβ6 antibody levels were detected in 51.8% of patients with UC and were significantly higher than those observed in patients with CD (*p* < 0.0001) and healthy controls (*p* < 0.0001), while no significant differences were found between the latter two groups. However, it should be noted that, at enrollment, the UC and CD cohorts differed significantly with respect to several baseline characteristics, particularly disease activity and the distribution of ongoing treatments, including mesalamine, immunosuppressants, and biologic therapies. Notably, anti-αvβ6 antibodies have also been proposed as predictive biomarkers for the future development of UC, as they may be detectable up to 10 years before disease onset (AUC 0.8), thereby opening new perspectives for preventive strategies in inflammatory bowel disease [77].

Abdominal pain represents a relevant potential therapeutic target that, despite its profound impact on quality of life and work productivity, remains insufficiently characterized in the literature and is frequently undertreated [78]. Its pathogenesis is complex and multifactorial, extending beyond active intestinal inflammation to include mechanisms such as peripheral and central sensitization, visceral hypersensitivity, altered brain–gut interactions, overlap with irritable bowel syndrome, dysbiosis or small intestinal bacterial overgrowth, psychological comorbidities including anxiety and depression, and genetic susceptibility. Importantly, abdominal pain often persists despite treat-to-target–driven escalation of immunomodulatory therapy and the achievement of conventional therapeutic goals, such as endoscopic remission. Although chronic opioid use is still common for symptom control, it is associated with substantial adverse effects and increased mortality and should therefore be avoided. Optimal management requires a multidisciplinary approach that integrates effective disease control within a treat-to-target framework with adjuvant pharmacological and non-pharmacological interventions, including dietary and psychological strategies.

Another critical future challenge is to broaden the external validity of RCTs for everyday clinical practice. Pragmatic trials, studies that occupy a middle ground between explanatory trials and purely observational real-world evidence, may offer the best solution [79]. They preserve randomization without rigid inclusion/exclusion criteria, strict standardized interventions, and outcome definitions, thereby generating findings that are more meaningful in routine clinical settings [80]. An additional advantage of pragmatic trials is that their outcomes tend to be patient-centered or health system-centered: they assess symptom control, quality of life, frequency of disease flares, healthcare resource utilization and cost-effectiveness [81]. By enrolling broader UC populations and evaluating interventions within routine care environments, pragmatic trials can help translate the T2T strategy from theoretical concept into practical approach.

The OPTIMISE study provides an example of pragmatic RCT investigating two T2T strategies in mild-to-moderate UC: one based on home-based FCP monitoring versus a purely symptom-based approach using PRO-2. In the interventional arm, treatment was optimized using monthly FCP measurements during active disease phases (quarterly during remission) together with PRO-2, while the reference arm relied only on PRO-2. No significant difference was observed in mucosal healing (MES = 0) at 12 months. However, the composite endpoint (MES = 0, RB = 0, and SF ≤ 1) at 12 months was achieved significantly more often in the interventional arm than the reference arm (effect size [ES]: 0.17, 95% CI 0.02–0.32; *p* < 0.05). A similar result was obtained for MES ≤ 1, RB = 0 and SF ≤ 1 (ES: 0.22; 95% CI 0.07–0.37; *p* < 0.05) [82]. These findings support the feasibility and benefit of tight biomarker-based monitoring.

Regarding cost-effectiveness, data on UC remain limited. Most studies highlight the lack of cost-effectiveness for biologics, mostly due to their high costs; additionally, cost inputs are country-specific, limiting the generalizability of conclusions. To support broader implementation, T2T will require dedicated economic research to inform clinical practice across different healthcare settings. Some preliminary evidence comes from a microsimulation analysis comparing T2T monitoring strategies in UC patients, which showed that combining symptom and biomarker monitoring yielded the highest Quality-Adjusted Life Year, although symptom-only monitoring was the most cost-effective in most modeled scenarios, underscoring the economic trade-offs in T2T approaches [56]. Research on this subject must certainly be implemented to provide clinicians with a solid foundation on which to build clinical practice aimed at reducing long-term risks related to the disease.

## 6. Conclusions

In conclusion, the definition of treatment targets in UC has progressively evolved into a more comprehensive definition of disease control that encompasses clinical, endoscopic, histologic, and patient-reported outcome domains. Notably, despite their conceptual appeal, more ambitious targets—such as disease clearance—may be challenging to implement in routine clinical practice (due to the need for repeated endoscopic and histologic assessments, resource limitations, and patient burden), and their application may be more feasible in selected patient populations and specialized centers, supporting a pragmatic and stepwise adoption rather than a universal strategy.

A “Shared Decision Making (SDM)” approach promoting active patients’ engagement and alignment on therapeutic targets, monitoring intensity, and treatment adjustments, may enhance the feasibility of T2T strategies in clinical practice [83,84,85].

Currently, the T2T paradigm advocates tight monitoring with biomarkers or non-invasive techniques such as ultrasound, alongside periodic endoscopic reassessments, to guide proactive treatment adjustment towards predefined targets.

However, it is crucial to acknowledge that only limited evidence currently supports this strategy in UC.

Further studies are needed to establish the long-term benefits and cost-effectiveness of implementing T2T in routine clinical practice.

## Data Availability

No new data were created or analyzed in this study.
